# Post-marketing surveilance study of creatine-guanidinoacetic acid safety in healthy adults

**DOI:** 10.3389/fnut.2024.1414308

**Published:** 2024-07-29

**Authors:** Marijana Ranisavljev, Nikola Todorovic, Valdemar Stajer, Sergej M. Ostojic

**Affiliations:** ^1^Applied Bioenergetics Lab, Faculty of Sport and PE, University of Novi Sad, Novi Sad, Serbia; ^2^Department of Nutrition and Public Health, University of Agder, Kristiansand, Norway; ^3^Faculty of Health Sciences, University of Pecs, Pécs, Hungary

**Keywords:** creatine, guanidinoacetic acid, homocysteine, adverse events, safety

## Abstract

A post-marketing surveillance study assessed the adverse events and possible risk of elevated homocysteine levels after the supplementation with creatine-guanidinoacetic acid mixture in apparently healthy adults. The participants were recruited through social media platforms and online discussion boards, with side effects and total plasma homocysteine (T-Hcy) levels evaluated regularly during a supplementation period of 6 months. Thirthy eight individuals (*n* = 38, 34.2% female) completed the evaluation period and were included in the final analyses. Serious side effects were absent. Two participants (5.3%) reported transitional nausea during the introductory weeks of the supplementation; no participants stopped the treatment. Baseline T-Hcy levels were 11.6 ± 3.1 μmol/L (95% confidence interval [CI], from 10.6 to 12.6). The intervention induced a mild reduction in T-Hcy levels across the monitoring period (*p* = 0.028), with T-Hcy levels after 1, 2, 3, and 6 months were 10.4 ± 3.0 μmol/L, 10.6 ± 2.9 μmol/L, 10.1 ± 2.7 μmol/L, and 9.3 ± 2.8 μmol/L, respectively. These findings suggest the overall tolerability of creatine-guanidinoacetic mixture in healthy adults, with homocysteine-increasing risk of no concern.

## Introduction

The co-administration of creatine and guanidinoacetic acid (GAA) has emerged as an innovative approach to potentially enhance energy metabolism and target tissues that are typically challenging to reach with conventional creatine interventions (1). This novel blend has been suggested to offer a more effective and safe alternative in experimental and clinical nutrition. Currently, over 100 nutritional supplements containing creatine and GAA are available on the international market (2), providing accessibility to the general public. The efficacy of the creatine-GAA mixture has been demonstrated in various non-clinical and clinical populations (3–5), with preliminary research indicating minimal disturbances in clinical enzymes and other safety biomarkers following a 28-day administration (6). However, uncertainty persists regarding the possibility of adverse events arising from prolonged supplementation with this novel product, which raises concerns among the general public. Given GAA’s potential to increase homocysteine production (7), an independent risk factor for several cardiometabolic diseases, it is crucial to evaluate whether the mixture affects circulating homocysteine levels in the general population. Therefore, the primary objective of this post-marketing surveillance study was to assess the real-life prevalence and severity of adverse events, as well as the potential risk of elevated homocysteine levels, following supplementation with the commercially available creatine-GAA product in apparently healthy adults.

## Methods

This study represents an open-label, non-controlled, prospective data collection to assess the safety of creatine-GAA under real-life conditions in a healthy population. Apparently healthy active adult men and women interested in using the creatine-GAA product (CreGAATine ™, Carnomed, Novi Sad, Serbia) were eligible for inclusion. All participants engaged in at least 150 min of moderate-intensity activity per week. The only exclusion criteria were contraindications to creatine-GAA as described in the summary of product characteristics (e.g., pregnancy, lactation, cardiovascular and kidney disease). Participants were recruited through social media platforms and online discussion boards, with data collected from individuals residing in various Central European countries, including Serbia, Croatia, Bosnia and Herzegovina, Montenegro, and Hungary, between August 2023 and March 2024. No participants received any compensation for taking part in the present study, except for receiving the creatine-GAA product at no cost. The study protocol included an initial meeting with a health professional and up to five follow-up visits, covering a supplementation period of 6 months. Observations included participants’ demographic parameters, the prevalence and severity of adverse events (collected via in-person and phone interviews), and total plasma homocysteine (T-Hcy) levels. Supplementation compliance was monitored by the number of product sachets consumed. Participant data were recorded using standardized case report forms; the forms underwent screening to ensure completeness and data plausibility before analysis, with any missing or implausible data discussed by the investigators. All adverse events occurring during the monitoring period were documented, regardless of their potential causal relationship to creatine-GAA supplementation. Participants were instructed to comply with the recommended intake of creatine-GAA product (e.g., one sachet administered in the morning and evening each day). Changes in T-Hcy levels during the surveillance were analyzed with one-way ANOVA, utilizing the Tukey test to compare differences between baseline values and individual follow-up time points.

## Results

**Figure 1:**
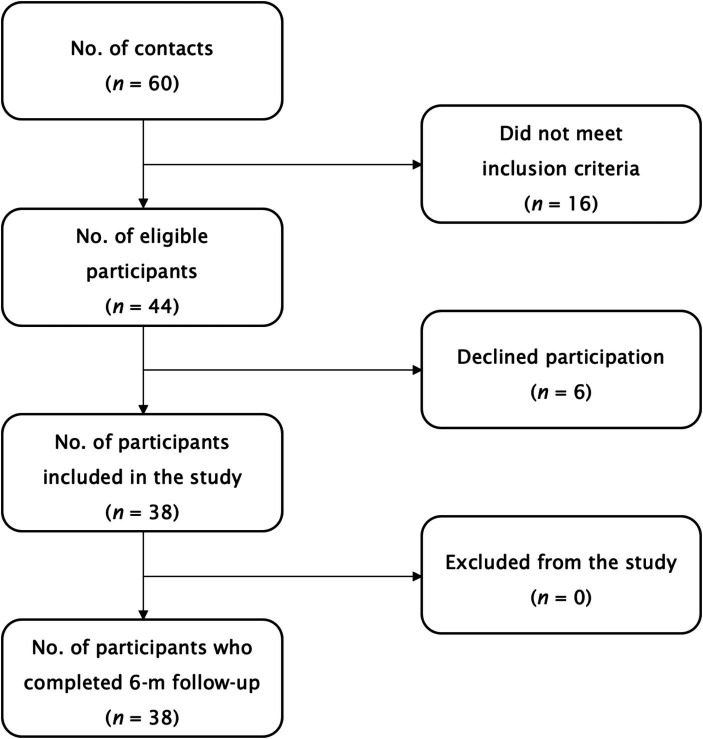
The participants flow during the study.

The participants flow during the study is illustrated in Figure 1. A total of 38 individuals (*n* = 38, 34.2% female; age aged 21–40 years, with a body mass index of 17–32 kg/m^2^) participated in the study, completed all follow-up visits, and were included in the final analysis. Treatment compliance was high at 91.4 ± 6.3% (95% confidence interval [CI]: 89.3–93.5). No serious adverse events, defined as events causing persistent or significant disability/incapacity, were reported. Three adverse events (7.9%) were disclosed: one participant (female, age 29) reported transient nausea related to the intervention, another participant (male, age 23) reported gastrointestinal disturbances after taking the evening dose of the supplement, and a third participant (male, age 31) reported a prostate issue at the final follow-up visit, not attributed to the supplementation. None of the participants discontinued supplementation due to adverse events.

**Figure 2:**
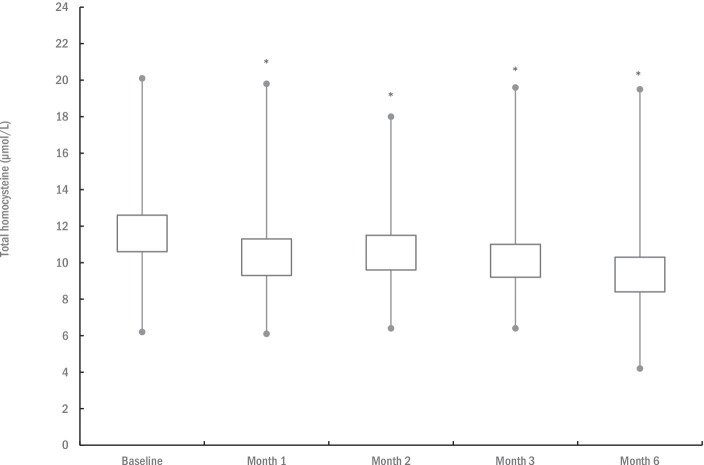
The changes in total plasma homocysteine levels during the study. The box illustrates the 95% confidence intervals, with lines extending to the minimum and maximum values. An asterisk (*) denotes significantly different mean values compared to baseline at *p* ≤ 0.05.

Changes in T-Hcy levels are depicted in Figure 2. The mean baseline T-Hcy levels were 11.6 ± 3.1 μmol/L (95% confidence interval [CI]: 10.6 to 12.6). One-way ANOVA revealed significant differences in T-Hcy levels across the five assessment points (*p* = 0.028), with mean T-Hcy levels significantly lower after 1 month (10.4 ± 3.0 μmol/L; *p* < 0.001), 2 months (10.6 ± 2.9 μmol/L; *p* = 0.002), 3 months (10.1 ± 2.7 μmol/L; *p* < 0.001), and 6 months of supplementation (9.3 ± 2.8 μmol/L; *p* < 0.001), compared to baseline levels. Initially, four participants (10.5%) exhibited hyperhomocysteinemia (≥ 15 μmol/L) at baseline, while this was observed in two participants (5.3%) at the 6-month follow-up.

## Discussion

This post-marketing surveillance study demonstrates that the commercially available product containing creatine and GAA (CreGAATine ™) is well tolerated in healthy adults, with no serious adverse events reported. Only a small proportion of participants (5.3%) experienced transient gut disturbances during the intervention. Creatine-GAA supplementation led to a mild decrease in circulating homocysteine levels, indicating no significant concerns regarding homocysteine elevation associated with this mixture. Our findings align with preliminary studies where the combination of creatine and GAA mitigated the rise in homocysteine levels (4, 6). This phenomenon may be attributed to creatine potentially modulating endogenous creatine biosynthesis, thereby lowering homocysteine production (8), or to the provision of methyl group donors in the supplement, such as folate, which facilitate the remethylation of homocysteine to methionine (9). Our study adds to the existing literature by evaluating real-life conditions with a robust sample size and repeated assessments of a critical biomarker. However, the relatively limited sample size, primarily composed of a male population, restricts our ability to identify potential gender-specific effects. Therefore, further research is needed to confirm the tolerability of the creatine-GAA mixture across more extensive and diverse populations, including the elderly and clinical cohorts, over longer exposure durations.

## Data availability statement

The raw data supporting the conclusions of this article will be made available by the authors, without undue reservation.

## Ethics statement

The studies involving humans were approved by the ethical approval was not required for post-marketing surveillance study. The studies were conducted in accordance with the local legislation and institutional requirements. The participants provided their written informed consent to participate in this study.

## Author contributions

MR: Data curation, Formal analysis, Investigation, Methodology, Writing – review & editing. NT: Data curation, Formal analysis, Investigation, Methodology, Writing – review & editing. VS: Conceptualization, Data curation, Investigation, Resources, Supervision, Validation, Writing – review & editing. SO: Conceptualization, Data curation, Formal analysis, Investigation, Methodology, Supervision, Visualization, Writing – original draft, Writing – review & editing.
